# Novel and Recurrent *MYO7A* Mutations in Usher Syndrome Type 1 and Type 2

**DOI:** 10.1371/journal.pone.0097808

**Published:** 2014-05-15

**Authors:** Weining Rong, Xue Chen, Kanxing Zhao, Yani Liu, Xiaoxing Liu, Shaoping Ha, Wenzhou Liu, Xiaoli Kang, Xunlun Sheng, Chen Zhao

**Affiliations:** 1 Ningxia Eye Hospital, Ningxia People’s Hospital, Ningxia, China; 2 Department of Ophthalmology, The First Affiliated Hospital of Nanjing Medical University, State Key Laboratory of Reproductive Medicine, Nanjing, China; 3 Department of Ophthalmology & Visual Sciences, The Chinese University of Hong Kong, Hong Kong, China; 4 Tianjin Medical University, Tianjin Eye Hospital, Tianjin Key Laboratory of Ophthalmology and Visual Science, Tianjin, China; 5 Department of Ophthalmology, Xinhua Hospital, Shanghai Jiao Tong University School of Medicine, Shanghai, China; 6 State Key Laboratory of Ophthalmology, Zhongshan Ophthalmic Center, Sun Yat Sen University, Guangzhou, China; National Cancer Institute, National Institutes of Health, United States of America

## Abstract

Usher syndrome (USH) is a group of disorders manifested as retinitis pigmentosa and bilateral sensorineural hearing loss, with or without vestibular dysfunction. Here, we recruited three Chinese families affected with autosomal recessive USH for detailed clinical evaluations and for mutation screening in the genes associated with inherited retinal diseases. Using targeted next-generation sequencing (NGS) approach, three new alleles and one known mutation in *MYO7A* gene were identified in the three families. In two families with USH type 1, novel homozygous frameshift variant p.Pro194Hisfs*13 and recurrent missense variant p.Thr165Met were demonstrated as the causative mutations respectively. Crystal structural analysis denoted that p.Thr165Met would very likely change the tertiary structure of the protein encoded by *MYO7A*. In another family affected with USH type 2, novel biallelic mutations in *MYO7A*, c.[1343+1G>A];[2837T>G] or p.[?];[Met946Arg], were identified with clinical significance. Because *MYO7A*, to our knowledge, has rarely been correlated with USH type 2, our findings therefore reveal distinguished clinical phenotypes associated with *MYO7A*. We also conclude that targeted NGS is an effective approach for genetic diagnosis for USH, which can further provide better understanding of genotype-phenotype relationship of the disease.

## Introduction

Usher syndrome (USH) is a group of autosomal recessively inherited disorders characterized by bilateral sensorineural hearing loss (SNHL) and a gradual retinal degeneration typified as retinitis pigmentosa (RP). Some patients with USH may also manifest vestibular dysfunction [1]. USH is estimated to be responsible for the majority of deaf-blindness in human (accounting for over 50%) [2], [3], with its prevalence ranging from 3.2 to 6.2 per 100,000 people in different populations [4], [5].

USH is clinically and genetically heterogeneous. Three major clinical subtypes have been described called USH type 1, type 2, and type 3. They are mainly distinguished by the severity and progression of the deafness and the presence of vestibular defects [6], [7]. Since the identification of the first USH-causative gene (*MYO7A*) in 1995 [8], by far, mutations in twelve genes have been implicated in the etiology of USH. USH type 1, accounting for 30% to 40% of total USH, is the most severe clinical form typified by profound congenital deafness, early onset of visual impairment (usually within the first decade of life), and vestibular dysfunction [9]. USH type 1 has been associated with nine genetic loci (USH1B-1H, 1J and 1K), among which six corresponding genes were identified including *MYO7A* (USH1B) [8], *USH1C* (USH1C) [10], *CDH23* (USH1D) [11], *PCDH15* (USH1F) [12], *USH1G* (USH1G) [13], and *CIB2* (USH1J) [14]. USH type 2, the most common form of USH, is presented with much less severer phenotypes than USH type 1 [9]. Typical clinical manifestations include normal vestibular functions, and moderate to severe congenital hearing impairments rather than totally deafness. RP usually occurs in the 2^nd^ decade. Three disease causing genes from distinct genetic loci have been reported, namely *USH2A* (USH2A) [15], *GPR98* (USH2C) [16], and *DFNB31* (USH2D) [17]. Patients with Usher type 3 are manifested by variable onset of progressive hearing loss, variable vestibular dysfunction, and also variable onset of visual impairment [3]. Mutations in *CLRN1* [18] have been pinpointed to cause USH type 3. Other than all above mentioned USH causative genes, the *PDZD7* gene encodes a protein which would interact with proteins encoded by *DFNB31* and *USH1C*, and may modify other Usher syndrome relevant mutations, we therefore have included *PDZD7* as another putative disease causative gene for Usher syndrome [19].

Mutations in *MYO7A* are the most common causes for patents with USH type 1 globally, accounting for 29% to 50% of total [5], [20], [21], [22], [23]. In the present study, we report three novel mutations and a recurrent one in *MYO7A* gene identified in three Chinese families with autosomal recessive USH type 1 and type 2 using targeted gene approaches. We have also identified genotype-phenotype correlation between *MYO7A* mutations and USH type 2, which, to the best of our knowledge, has not been previously characterized in details.

## Materials and Methods

### Participants and Clinical Investigations

This study was approved by the Ethics Committee on Human Research of the People’s Hospital of Ningxia Hui Autonomous Region, and conformed to the tenets of the Declaration of Helsinki. Written informed consents were obtained from all participants. Seven individuals, including four patients and three unaffected family members, of three unrelated families were recruited from Ningxia Hui Autonomous Region. Each participant underwent detailed pure tone audiometry testing and ophthalmic examinations, including best-corrected visual acuities (BCVAs) measurements, slit-lamp biomicroscopy, intraocular pressure (IOP) test, funduscopy, visual field (VF) test, and electroretinography (ERG) test. Detailed vestibular tests were further conducted on patients USH01-III:1 and USH01-III:2 as detailed previously [24]. Additionally, 100 controls over 60-year-old free of retinal dystrophies or other major eye diseases were included. Peripheral blood samples were obtained from all participants. Genomic DNA was extracted from leukocytes using a QIAmp DNA Blood kit (Qiagen, Valencia, CA) per the manufacturer’s instructions.

### Targeted Gene Capture and Next-generation Sequencing

Two similar targeted sequence capture microarrays were used in this study. Microarray #1, the RDs189-Array, has been previously described and validated [25]. This chip was designed to capture the coding sequences with at least 100 base pairs (bps) of flanking intronic sequences, and 5′- and 3′- untranslated regions (UTR) of 179 genes associated with HRDs and 10 candidate genes including all USH-related genes. Probes of 250–300 bps were designed accordingly for sequence capture. Targeted gene approach using the RDs189-Array was performed on the two affected members in family USH01, USH01-III:1 and USH01-III:2. Microarray #2 was a commercial sequence capture array (BGI-Shenzhen, Shenzhen, Guangdong, China) that could capture the coding sequences of 316 genes with all known USH causative genes included. Patient USH02-II:1 and USH03-II:1 were screened using microarray #2. Information of the targeted genes of the commercial array has been previously described [26]. Sequence capture, enrichment, elution, and NGS were conducted in collaboration with the BGI-Shenzhen as previously stated [27].

### Bioinformatics Analysis and Mutation Validation

The NCBI human reference genome (NCBI build 37.1) was obtained from the UCSC database (http://hgdownload.cse.ucsc.edu/goldenPath/hg19/bigZips/). Bioinformatics analysis was performed as described previously [25]. Briefly, reads were aligned to the reference genome using Short Oligonucleotide Alignment Program (SOAP, http://soap.genomics.org.cn) for SNP analysis [28], and Burrows-Wheeler Aligner (BWA, http://bio-bwa.sourceforge.net/) for Indel detection [29]. Coverage and depth calculations were only performed on aligned reads. All detected variants were further filtered against the following 5 SNPs databases, including dbSNP137 (http://hgdownload.cse.ucsc.edu/goldenPath/hg19/database/snp137.txt.gz.), HapMap Project (ftp://ftp.ncbi.nlm.nih.gov/hapmap), 1000 Genome Project (ftp://ftp.1000genomes.ebi.ac.uk/vol1/ftp), YH database (http://yh.genomics.org.cn/), and Exome Variant Server (http://evs.gs.washington.edu/EVS/). Variants found homozygous in those databases or with minor allele frequency (MAF) >0.01 were filtrated. Datasets of NGS were deposited in National Institutes of Health (NIH) Short Read Archive (http://www.ncbi.nlm.nih.gov/sra). Sanger sequencing was subsequently performed for validation of variants in all USH causative genes and prevalence test in 100 unrelated controls, using a previously defined protocol [30]. Primer information was detailed in **Table S1**.

### *In silico* Analyses

Vector NTI Advance 11 software (Invitrogen, Grand Island, NY) was applied to evaluate the evolutionary conservation of the mutated amino acids through alignment of the *MYO7A* orthologous protein sequences of the following species: *Homo sapiens* (ENSP00000386331), *Pan troglodytes* (ENSPTRP00000007055), *Canis lupus familiaris* (ENSCAFP00000007721), *Bos taurus* (ENSBTAP00000005191), *Sus scrofa* (ENSSSCP00000015796), *Mus musculus* (ENSMUSP00000102745), *Gallus gallus* (ENSGALP00000001044), *Danio rerio* (ENSDARP00000083378), *Drosophila melanogaster* (FBpp0080282), and *Caenorhabditis elegans* (T10H10.1). The possible impacts of the mutation were indicated by three online software, including SIFT Human Protein DB (http://sift.bii.a-star.edu.sg/), PolyPhen-2 (Polymorphism Phenotyping, version 2; http://genetics.bwh.harvard.edu/pph2/) [31], and CONDEL (http://bg.upf.edu/condel/analysis). The effect of alteration of splice site was predicted by Human Splicing Finder version 2.4.1 (http://www.umd.be/HSF/), NetGene2 sever (http://www.cbs.dtu.dk/services/NetGene2/), Splice Site Prediction by Neural Network (http://www.fruitfly.org/seq_tools/splice.html), and SROOGLE (http://sroogle.tau.ac.il/). SWISS-MODEL online server was used to predict the crystal structures of the wide type and mutant proteins [32], [33]. Predicted structures were displayed using PyMol software (version 1.5).

## Results

### Clinical Manifestations

**Figure 1:**
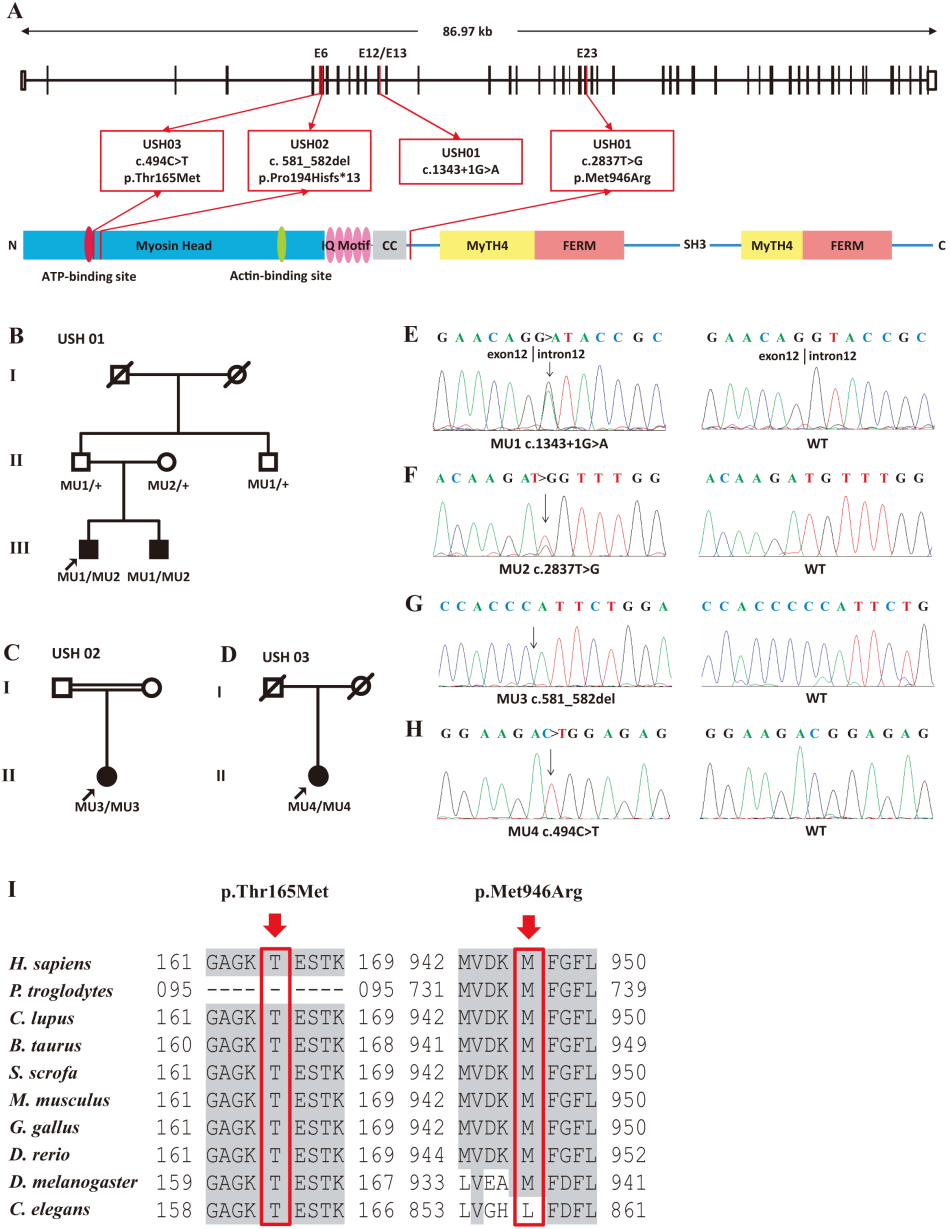
Family pedigrees, DNA chromatogram sequences, and sequence alignment of the identified mutational spots. (A) Schematic representation of the relative linear location of all four *MYO7A* mutations identified in the present study in context of genome structure (upper) and protein structure (below). (B–D) Pedigrees of families USH01 (B), USH02 (C), and USH03 (D). Filled and empty symbols represent affected and unaffected individuals, respectively. Probands (USH01-III1, USH02-II1, and USH03-II1) are indicated by arrows. Consanguinity exits in family USH02. (E–H) DNA sequencing profiles for the four identified disease-causing mutations (left panel) and the corresponding wide type forms (right panel). Biallelic heterozygous c.1343+1G>A (E, MU1) and c.2837T>G (F, MU2, p.Met946Arg) mutations are identified as disease causing mutations for family USH01. Homozygous c.581_582del (G, MU3, p.Pro194Hisfs*13) and c.494C>T (H, MU4, p.Thr165Met) mutations are indicated as disease causative for family USH02 and USH03, respectively. (J) The arrows indicate that the Thr165 and Met946 residues are evolutionary highly conserved among multiple species.

**Figure 2:**
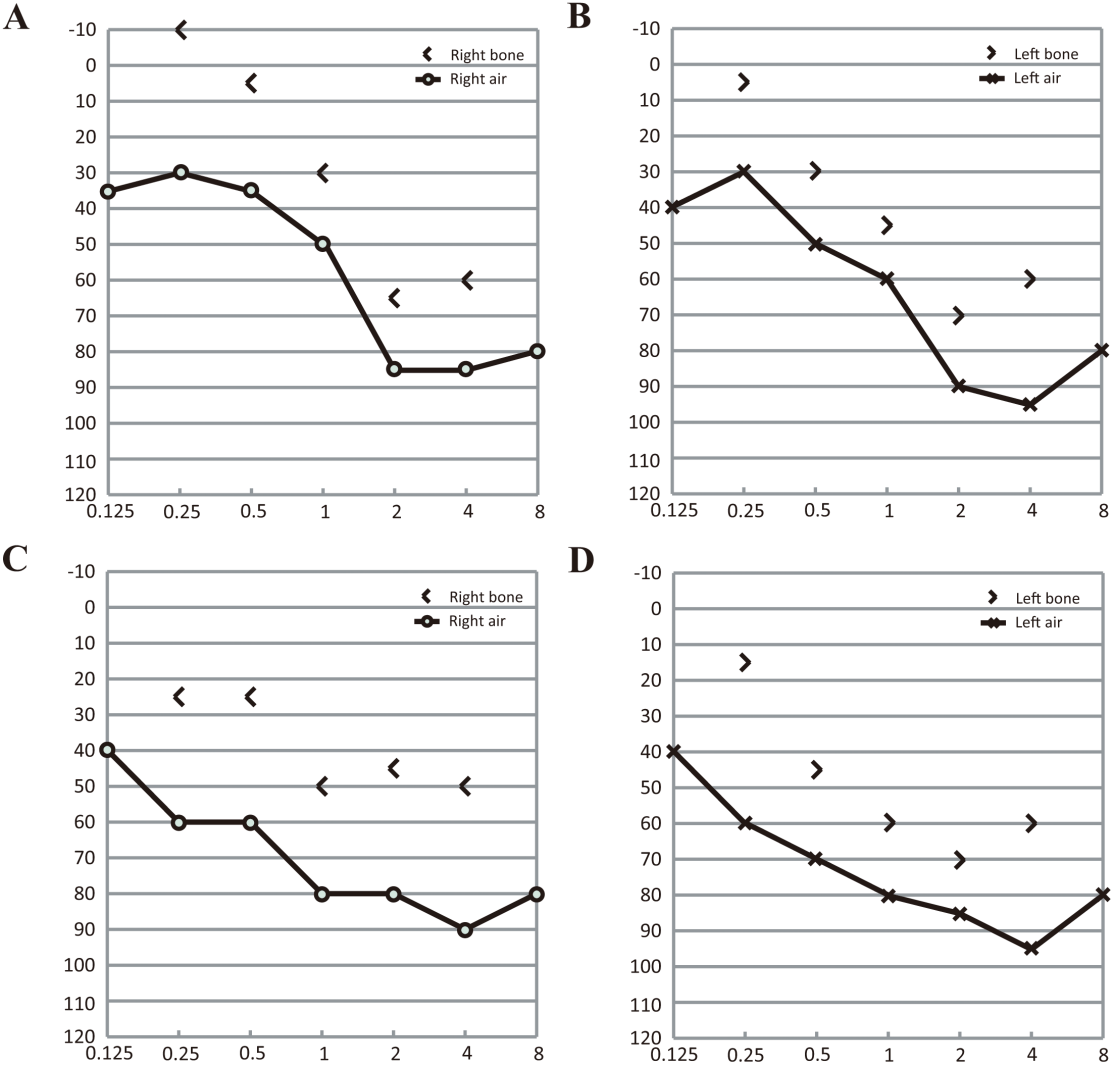
Pure tone audiometry results of the two patients in family USH01. (A–B) Pure tone audiometry results of patient USH01-III1 show moderate bilateral hearing impairments. (C–D) Moderate to severe bilateral sensorineural hearing loss is revealed in patient USH01-III2.

**Figure 3:**
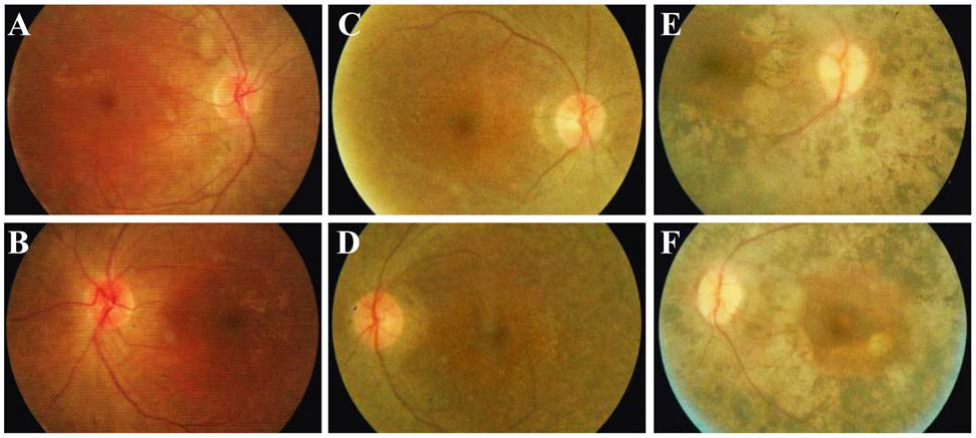
Fundus photographs of the included patients. (A–B) Fundus photographs of the 24-year-old proband of family USH01 reveal attenuation of retinal vessels, pallor of optic disc, and pigmentary migration, indicating an atypical RP fundus. Macular region is not affected in both eyes. (C–D) Fundus appearance of patient USH01-III2 (22-year-old) is similar to that of patient USH01-III1. Attenuated retinal vessels and pale optic disc are observed. Macular region is also preserved. (E–F) Typical RP fundus is shown by patient USH03-II1, including bone spicule-like pigmentation, retinal vascular attenuation, pallor of optic disk, and chorioretinal atrophy. Macular degeneration is observed in this patient.

Four affected members and three unaffected individuals from 3 families with USH were included in this study (**Figure 1B–1D**). The clinical manifestations of the affected members were detailed in **Table 1**. In family USH01, the parents of the two affected patients were asymptomatic. The 24-year-old proband, USH01-III:1, was found to have hearing problems since childhood. Pure tone audiometry testing revealed bilateral moderate sensorineural hearing impairment in the proband, which were 56.67 and 66.67 dB for the right and left ear, respectively (**Figure 2A, 2B**). His speech was normal and the hearing impairment was non-progressive. No vestibular defect was detected. He developed night blindness at 15-year-old age. RP characteristics were indicated by fundus photo, including vessel attenuation, pallor of optic disc, and pigmentary migration (**Figure 3A, 3B**). VF was significantly constricted to tubular vision, and ERG was diminished in both rod and cone responses. His current BCVA was 0.8 in both eyes. Macular region was relatively preserved according to his fundus appearance and OCT results. The anterior segment and IOP were also normal. The disease symptoms of his younger brother were a bit severer. He had non-progressive moderate to severe hearing loss (73.33 dB and 78.33 dB for the right and the left ear, respectively, **Figure 2C, 2D**), and slight language barrier. Similar to his brother, his RP onset age was 13, and he was also manifested with typical RP symptoms (**Figure 3C, 3D**). Since the RP onset ages for the two patients were 15 and 13 years of age, both of which were in accordance with the typical clinical manifestations presented by Usher syndrome type 2, we therefore concluded that the two patients from family USH01 displayed USH type 2 phenotypes.

**Table 1:** Clinical Manifestations of Patients Carrying *MYO7A* Mutations.

	USH01-III:1		USH01-III:2		USH02-II:1		USH03-II:1	
	O.D.	O.S.	O.D.	O.S.	O.D.	O.S.	O.D.	O.S.
**Genotype (** ***MYO7A*** **)**								
*Type of Mutation*	Biallelic		Biallelic		Homozygous		Homozygous	
*Nucleotide Variation*	c.[1343+1G>A];[2837T>G]		c.[1343+1G>A];[2837T>G]		c.581_582del		c.494C>T	
*Amino Acid Change*	p.[?];[Met946Arg]		p.[?];[Met946Arg]		p.Pro194Hisfs*13		p.Thr165Met	
*Novel or Recurrent*	Novel		Novel		Novel		Recurrent	
**Frequency in Control Alleles**	0/200		0/200		0/200		0/200	
**Accession Numbers in National Institutes of Health Sequence Read Archive**								
*BioProject*	SRP040003		SRP040003		SRP040003		SRP040003	
*BioSamples*	SRS579268		SRS579270		SRS569215		SRS579266	
*Runs*	SRR1200449		SRR1200450		SRR1200411		SRR1200446	
**Age (years)/Se**x	24/M		22/M		50/F		60/F	
**Walking Age**	12-month		12-month		20-month		24-month	
**Onset Age for Hearing Impairments**	since born		since born		since born		since born	
**Onset Age for Visual Defects**	15-year-old		13-year-old		7-year-old		6-year-old	
**BCVAs (logMAR)**	0.8	0.8	0.6	0.7	FC	FC	HM	HM
**IOP (mmHg)**	14	13.8	13.2	14.5	14.5	18.5	17.5	16.5
**Fundus Appearance**								
*Macular Degeneration*	No	No	No	No	Yes	Yes	Yes	Yes
*Optic Disk*	Pale	Pale	Pale	Pale	Pale	Pale	Pale	Pale
*Artery Attenuation*	Yes	Yes	Yes	Yes	Yes	Yes	Yes	Yes
*Pigment Deposits*	Yes	Yes	Yes	Yes	Yes	Yes	Yes	Yes
**Visual Field**	TV	TV	TV	TV	NA	NA	NA	NA
**Electroretinography**	Diminished	Diminished	Diminished	Diminished	NA	NA	NA	NA
**Clinical Diagnosis**	USH type 2		USH type 2		USH type 1		USH type 1	

Abbreviations: USH: usher syndrome; O.D.: right eye; O.S.: left eye; SD: splice site mutation; M: male; F: female; BCVA: best-corrected visual acuities; FC: finger count; HM: hand move; IOP: intraocular pressure; TV: tubular vision; NA: not available.

Unlike family USH01, patients USH02-II:1 and USH03-II:1 are presented with typical USH type 1 phenotypes. Both patients suffered from severe bilateral hearing loss and severe speech problems since childhood. They also had vestibular dysfunctions. RP developed within the first decade of their life. Typical RP fundus and other ophthalmic examination results were revealed in both patients (**Figure 3E, 3F**). The detailed clinical information was listed in **Table 1**.

### Targeted Gene Approach

As aforementioned, targeted gene approach was performed on patients USH01-III:1, USH01-III:2, USH02-II:1, and USH03-II:1. The detailed results underlying NGS are discussed in **Table S2**. Generally, an over 98.54% coverage of target region was processed by each sample, and the rate of nucleotide mismatch for each sample was equal to or below 0.25%. A total of 8939 variants were initially disclosed in all three families, including 8041 SNPs and 898 Indels. We deposited our dataset in NIH Short Read Archive, and have provided the accession numbers of BioProject, Biosamples, and Runs for each individual in **Table 1**. The identified variants were first annotated to the 5 SNPs databases. Variants found homozygous in any of the 5 databases or with MAF>0.01 were filtered. Synonymous and non-coding variants (with exception of intronic variants within 10 bp flanking exons) were further removed. Remaining variants in all USH causative genes (as listed in **Table S3**) were subsequently confirmed by Sanger sequencing, and also tested in all attainable family members within each individual family and 100 unrelated negative controls. In all, four putative pathogenic variants in *MYO7A* gene were identified in the three individual families, of which three were novel, and one was recurrent. All four detected mutations were absent in 100 unrelated negative controls.

### Novel Biallelic *MYO7A* Mutations in Family USH01 with USH Type 2

Novel biallelic heterozygous mutations, *MYO7A* c.[1343+1G>A];[2837T>G], were detected in the two patients from family USH01 affected with USH type 2 (**Figure 1A, 1E–1F**). All mutations identified in this manuscript were described according to the guidelines summarized on the Mutation Nomenclature Homepage at the HGVS website (http://www.hgvs.org/mutnomen/). The former one, c.1343+1G>A, refers to a G to A substitution at the first nucleotide of the intron 12, the absolutely conserved donor splice site, which is right adjacent to the coding nucleotide numbered as c.1343, the last nucleotide of exon 12. The consensus splice site was eliminated according to results from all four online prediction websites, while a potential novel one would be produced over 300 bps away from the previous site. The latter mutation would result in a substitution of methionine for arginine at condon 946 (p.Met946Arg), which was found to be deleterious as revealed by all three online prediction software, including Polyphen (0.942, possibly damaging), SIFT (0.00, deleterious), and CONDEL (0.678, deleterious). Conservational analysis further indicated that this position was highly conserved among all mammalian animals (**Figure 1I**). Cosegregation analysis revealed that the two patients inherited the c.1343+1G>A mutation paternally and the p.Met946Arg mutation maternally.

### Mutation Analyses in Family USH02 and USH03 with USH Type 1

**Figure 4:**
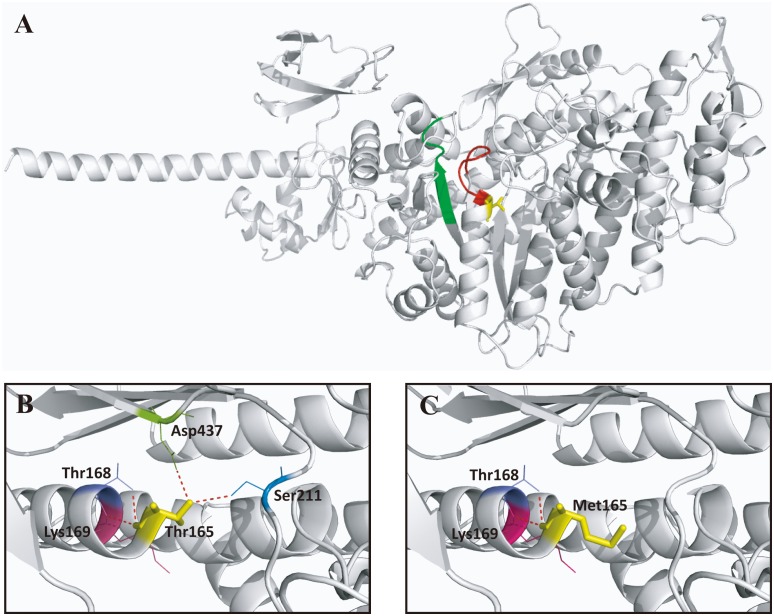
Structural modeling of myosin-VIIa. (A) Overview of the predicted structure of myosin-VIIa (3–769) covering the majority of the myosin head-like domain (1–729) and the first IQ motif (745–765). The ATP binding site (158–165) and the actin binding site (632–639) are included and are colored as red and green, respectively. The mutational spot identified in patient USH03-II:1 (Thr165) located in the ATP binding site is indicated in yellow. (B) A close view of the residue 165 highlighting the wide type amino acid threonine, the generated hydrogen bonds (in red), and their interacted amino acids, including Thr168, Lys169, Ser211, and Asp437. (C) Two previously indicated hydrogen bonds are eliminated due to the change of the wide type threonine into the mutant methionine.

In the consanguineous family USH02, a novel homozygous variant, *MYO7A* c.581_582del, was identified in the patient (**Figure 1G**). The framshift mutation indicated a deletion of two coding nucleotides at c.581 and c.582. This deletion was a frameshift mutation with introduction of a premature termination codon (PTC), and therefore, the protein change is theoretically p.Pro194Hisfs*13, a truncated protein with 205 amino acid. However, the truncated proteins may not exist *in vivo* due to NMD. The sporadic case from family USH03 was found to carry a recurrent homozygous variants in *MYO7A*, c.494C>T [34], which would lead to the amino acid change from threonine to methionine at condon 165 (p.Thr165Met) (**Figure 1H**). Severe functional impact of this variant was indicated by all three online prediction software. That was 1.000 (possibly damaging) for Polyphen, 0.00 (deleterious) for SIFT, and 1.000 (deleterious) for CONDEL. Consistently, this mutational spot was highly conserved among all species analyzed by multiple protein sequences alignment (**Figure 1I**). We conducted structural modeling of the myosin-VIIa (residues 3–769), the protein encoded by *MYO7A* gene, on the basis of the crystal structure of Q02440, the motor domain of the chicken myosin-Va showing the sequence identity of 39.51% with the human myosin-VIIa [35]. The model built revealed that the wild type Thr165 located in the ATP binding site (158–165) generated four hydrogen bonds and interacted with four amino acids including Thr168, Lys169, Ser211, and Asp437 (**Figure 4A, 4B**). The substitution of threonine to methionine at residue 165 resulted in the elimination of hydrogen bonds between residue 165 and Ser211, and between residue 165 and Asp437 (**Figure 4C**). The elimination of the two hydrogen bonds, which link an α-helix with β-sheets, would potentially change the tertiary structure of the protein. Thus, we conclude that the variants identified are very likely the disease-causing mutations of the corresponding family.

## Discussion

Mutations in *MYO7A* gene have long been implicated in the disease etiology of USH type 1. In the present study, three novel mutations and a recurrent one were revealed as disease-causing in three unrelated families. Noteworthy, in a Chinese family with USH type 2, novel biallelic heterozygous mutations in *MYO7A* were identified namely c.[1343+1G>A];[2837T>G]. The two mutations cosegregated with disease phenotypes and were absent in 100 normal controls. To our knowledge, *MYO7A* mutations have rarely been correlated with USH type 2. Only Bonnet et al has identified biallelic *MYO7A* mutations in a sporadic Caucasian patient [36], but with no clinical data provided in their paper in addition to diagnosis. In our manuscript, we have summarized the clinical findings of a Chinese family with USH type 2, in which we identified novel biallelic *MYO7A* mutations. Therefore, we have provided sufficient analysis of genotype-phenotype correlation between *MYO7A* mutations and USH type 2, which, to our understanding, has not been previously characterized in details. Located on 11q13.5, the *MYO7A* gene yields a transcript of 7.46 Kb encompassing 49 exons. The encoded protein, myosin-VIIa, is an actin-based motor protein that plays an important role in intracellular transport, endocytosis, and intercellular adhesion [37]. Expressions of the myosin-VIIa protein in the cochlear hair cells, retinal photoreceptors, and retinal pigment epithelium (RPE) have been detected [38], [39]. Although disagreements still exist regarding the subcellular localization of the myosin-VIIa protein, most studies support the association between the protein and the connecting cilium [40], [41], [42]. In rod photoreceptors, myosin-VIIa resides at the connecting cilium and functions in transporting rhodopsin to the rod outer segment (OS) discs [43], [44]. In RPE, that protein localizes to the apical side [38], [39], and deals with the proper localization of melanosomes to the RPE microvilli [45], [46] and phagosome motility [47].

Since first identification in 1995, mutations in *MYO7A* gene have been reported to be implicated in causing a wide phenotypic spectrum, including USH type 1 [8], an atypical form of USH mimicking USH type 3 [34], two types of nonsyndromic deafness: autosomal dominant nonsyndromic deafness-11 (DFNA11) [48] and autosomal recessive nonsyndromic deafness-2 (DFNB2) [49], [50], and even Leber congenital amaurosis [51]. Based on the great clinical and genetic heterogeneities, there has been hypothesis concerning the relationship between the localizations of the mutations and the diverse clinical phenotypes. The 2215 amino acids protein myosin-VIIa presents the following domains: a myosin head-like domain (1–729), which contains the crucial ATP-binding site (158–165) and actin-bind site (632–639), five IQ motifs (IQ1: 745–765, IQ2: 768–788, IQ3: 791–811, IQ4: 814–834, IQ5: 837–857), a coiled-coil region (858–935), two MyTH4 domains (1017–1253, and 1747–1896), two FERM domains (1258–1602, and 1902–2205), and an SH3 domain (1603–1672) [5].

Three of the four identified mutations are located within the myosin head-like domain. The recurrent missense mutation detected in patient USH03-II:1, p.Thr165Met, will lead to the amino acid change from the polar threonine to a nonpolar methionine. The Thr165 residue is located in the highly conserved ATP binding pocket region (161–165). Therefore, this mutation would obstruct the binding between the ATP and the protein, thus disturbing the motor function and preventing head movement. In addition, the mutational change from threonine to methionine would eliminate two hydrogen bonds. The two hydrogen bonds, which link an α-helix with β-sheets according to the predicted crystal structure, are generally critical for the tertiary structure of the protein. Thus, the elimination of the two hydrogen bonds would potentially change the tertiary structure of the protein, and further disturb the internal interactions and reduce the stability of the protein. Noteworthy, one of the two eliminated hydrogen bonds was between resides Thr165 and Asp437, the latter of which has significance in the disease mechanism of Usher syndrome. For instance, the mutation p.Asp437N has previously been reported in patients with USH type 1 [52]. Residue Asp437 is located within the switch II loop of the myosin head-like domain, and is equivalent to residue Asp463 in chicken smooth muscle myosin and residue Asp454 in Dictyostelium. Residue Asp463 in chicken is recognized to form a hydrogen bond to the magnesium ion which coordinates the b- and g-phosphates of ATP [52], [53], and a substitution in Dictyostelium, p.Asp454Ala, would abolish actin-activated Mg21ATPase activity thus inhibiting the myosin from moving actin filaments [52], [54]. The p.Pro194Hisfs*13 identified in patient USH02-II:1 was also located within the myosin head-like domain. This novel frameshift variant would cause a PTC, and thus leading to nonsense mediated mRNA decay (NMD) [55] or the generation of a truncated protein containing 205 amino acids. However, the truncated proteins may not exist *in vivo* due to NMD.

The c.1343+1G>A affects a consensus donor splice site, which is essential for splicing of the intron 12. Similarly, multiple splice site mutations of the *MYO7A* gene have been reported previously [36], [56], [57], and two of those mutations were proved to alter the natural splicing through hybrid minigene assays [58]. In our case, in an attempt to understand the possible protein change resulted from c.1343+1G>A, we applied four online prediction software to evaluate the effect of c.1343+1G>A on splicing. As expected, all four programs similarly predicted that the DNA variant, c.1343+1G>A, would abolish the original splice donor site, resulting in the insertion of the beginning of intron 12 until a PTC, TAA, located at c.1343+50 to c.1343+52 in intron 12. Thus, the c.1343+1G>A allele is expected to lead to a NMD of the corresponding mRNA, which is not detectable by minigene assay, or, less likely, result into a truncated *MYO7A* proteins, p.Ser448Argfs*18.

Another mutation identified in patients USH01-III:1 and USH01-III:2 was a missense one, p.Met946Arg. That generates the amino acid change from the nonpolar methionine to an alkaline amino acid arginine at the highly conserved codon 946 between coiled coil region and the first MyTH4 domain. The two patients from family USH01 met the diagnostic criteria for USH type 2. The proband suffered from non-progressive moderate hearing impairments since childhood, while his younger brother had moderate to severe hearing loss and obstacles in communication. Ophthalmic examinations reveal RP phenotypes, including night blindness occurred in the 2^nd^ generation, tubular vision, and RP fundus. The vestibular reflection of the two patients was normal. The Met946 residue avoids the important functional domains of myosin-VIIa, and that may help explain the atypical phenotypes. But that is not exclusive since previous reports have revealed mutations in near sites (p.Gly955Ser) in patients with USH type 1 [49].

Similar to our findings in the present study, previous study has also reported the identification of biallelic heterozygous *MYO7A* mutations, including p.Ala457Val and p.Lys269del, in a sporadic patient with USH type 2 [36]. Both residues Ala457 and Lys269 are located within the myosin head-like domain, and of note, the location of p.Ala457Val is quite close to that of mutation p.Ser448Argfs*18 identified in our study, which could possibly explain for the USH type 2 phenotypes they caused. USH type 2 are associated with three genes, namely *USH2A*, *GPR98*, and *DFNB31*, implying the potentially complicated genotype-phenotype correlations for USH type 2. Mutations in *USH2A* account for over 70% of cases affected with USH type 2. In particular, many patients carrying mutations in *USH2A* showed moderate to severe SNHL with no vestibular defects and progressive retinitis pigmentosa [15], [59], all of which are very similar to the phenotypes observed in the present family USH01, and thus further confirm the clinical diagnosis of USH type 2 in this family. In addition, mutations in *USH2A* have been reported in causing nonsyndromic RP and nonsyndromic deafness [60], further indicating the clinical heterogeneity for USH causative mutations.

USH shows great clinical and genetic heterogeneities. Sometimes, it would be difficult for clinicians to make proper clinical diagnosis due to the atypical manifestations. Molecular diagnosis would definitely help with clinical diagnosis, and acts as the basis of prenatal testing and gene therapy. However, it would also be a huge job for mutation analysis using Sanger sequencing. At this point, our study proves that targeted gene approach is an efficient and economic tool in molecular diagnosis.

In conclusion, we here report biallelic novel heterozygous mutations in *MYO7A* gene leading to USH type 2 in a Chinese family. The genotype-phenotype correlation is discussed but the underlying mechanism needs further elucidations. Our study extends the spectrums of *MYO7A* mutations and its related clinical phenotypes. We also prove targeted gene approach as a valuable tool in molecular diagnosis.

## Supporting Information

Table S1 **Variants post filtration and primers used for mutation validation.**
(XLSX)

Table S2 **Overview of data production.**
(XLSX)

Table S3 **All variants in USH causative genes identified in the three families investigated.**
(XLSX)
